# Patient-Reported Social Impact of Molecularly Confirmed Retinitis Pigmentosa

**DOI:** 10.3390/jcm14093229

**Published:** 2025-05-06

**Authors:** Nina Zehe-Lindau, Birgit Lindau, Heidi Stöhr, Bernhard H. F. Weber, Georg Spital, Ulrich Kellner

**Affiliations:** 1 Rare Retinal Disease Center, AugenZentrum Siegburg, MVZ Augenärztliches Diagnostik- und Therapiezentrum Siegburg GmbH, Europaplatz 3, 53721 Siegburg, Germany; ninaz0412@gmail.com (N.Z.-L.); b.lindau@osg.de (B.L.); 2 RetinaScience, Postfach 301212, 53192 Bonn, Germany; 3 Institute of Human Genetics, University of Regensburg, Franz-Josef-Strauss-Allee 11, 93053 Regensburg, Germany; heidi.stoehr@klinik.uni-regensburg.de (H.S.); bweb@klinik.uni-regensburg.de (B.H.F.W.); 4 Institute of Clinical Human Genetics, University Hospital Regensburg, Franz-Josef-Strauss-Allee 11, 93053 Regensburg, Germany; 5 Augenzentrum am St. Franziskus-Hospital Münster, Hohenzollernring 74, 48145 Münster, Germany; georg.spital@augen-franziskus.de

**Keywords:** retinitis pigmentosa, quality of life, patient-reported impact, ophthalmologic care, social care, anxiety, financial burden

## Abstract

**Objectives**: To evaluate the patient-reported impact of retinitis pigmentosa (RP) in a large patient cohort to identify relevant disease-related disadvantages as key aspects for the improvement of ophthalmic and social care. **Methods**: Consecutive patients with molecularly confirmed RP older than 18 years of age were identified in two tertiary care centers in Germany. Patients were contacted to participate in an anonymized patient query regarding the impact of RP on their vocational training, professional career, and social, familial, and personal life, as well as their experience with ophthalmologic care. **Results**: Out of 241 contacted patients, 162 responded (67.2%; 52.5% female, 67.3% younger than 50 years of age). While the impact of RP on vocational training was limited, professional careers were frequently disrupted with early retirement rates of 39.8% (≥40 years of age) to 50% (≥50 years of age). Most respondents felt restricted in their participation in public life (66.3%). One-fifth complained about financial restrictions; however, one-third of this number did not obtain financial help. A negative impact on familial life (21.4%) was less frequently reported compared to personal impairments, especially anxiety (74.5%) and depression (24.2%). Most respondents considered their ophthalmic care as adequate; however, a delayed diagnosis (≥two years: 28.6%) and initial misdiagnosis (24.0%) were frequent. Insufficient psychological support was the major complaint about professional care. **Conclusions**: RP affects the lives of patients in multiple aspects. Ophthalmic and social care providers should focus on the acceleration of the diagnostic process, as well as easy access to financial assistance and psychological support, as key areas for improvement. Improvements in these areas are expected to reduce challenges for patients. They should have an impact on rehabilitation, participation in public life, and quality of life.

## 1. Introduction

Inherited retinal dystrophies (IRDs) are the leading cause of blindness in the working-age population (16–64 years) and represent a significant cause of childhood blindness (0–15 years) in industrialized countries [1,2,3,4,5,6].

Retinitis pigmentosa (RP) accounts for approximately 46% of IRD in Germany [7]. RP presents with varying clinical courses and ages of onset, though most patients report the onset of symptoms by age 15 [8]. Functional impairments affect daily life in multiple ways: night blindness limits independent mobility, especially during winter; visual field defects restrict activities such as driving, operating machinery, using stairs, or working at heights; and reduced visual acuity impacts educational and occupational performance. These disabilities often hinder proper education, increase the risk of being unable to pursue desired professions, or lead to job loss. Childhood-onset IRD has been shown to reduce lifetime income by approximately one-third [9,10,11] and raise personal healthcare expenses [12,13].

Visual impairments, compounded by financial challenges, negatively affect patients’ social and family lives, frequently leading to anxiety and depression [14,15]. Caregivers of individuals with RP are similarly impacted, experiencing declines in mental health and financial stability [16]. These detrimental effects on quality of life are often exacerbated by delays in diagnosis and gaps in ophthalmologic and psychological care—issues commonly cited by patients.

Recent studies have provided extensive patient-reported experiences with IRDs, including from the United Kingdom, Ireland [14], and Germany [17], as well as Canada and the United States [18]. However, patient selection criteria have varied considerably across these studies, often encompassing broader IRD populations without consistent genetic confirmation.

The present study aimed to address this gap by conducting a large, anonymous survey focused exclusively on patients with molecularly confirmed RP. All participants had their clinical diagnoses established at one of two specialized centers in Germany, ensuring a well-defined and homogeneous study population.

## 2. Materials and Methods

The aim of this study was to reach a large patient cohort using a concise, anonymous questionnaire to maximize the response rate. A consecutive series of molecularly confirmed cases of non-syndromic or syndromic RP was identified by searching separate databases at two separate specialized centers by two of the authors (G.S. and U.K.). Patients were eligible for inclusion if they:

- had at least one pathogenic or likely pathogenic variant in autosomal dominant or X-linked RP, or two pathogenic or likely pathogenic variants in a known gene for autosomal recessive RP;
- had been examined at one of the centers at least once between January 2018 and January 2024;
- were at least 18 years old at the time of the survey;
- and resided in Germany.

Based on these search parameters, 260 patients were identified who met the inclusion criteria. The majority of these patients were of Caucasian ethnicity. Clinical assessments and molecular genetic confirmation were conducted as previously described [19,20]. This study received ethics approval from the respective ethics committees of the North Rhine (ID: 2024037, 27 February 2024) and Westphalia-Lippe (ID 2024-234-b-S, 9 April 2024) medical associations.

The questionnaire was designed based on previously reported patient concerns and consisted of nine structured questions with predefined answer options (see Supplementary Materials). To ensure anonymity, age was recorded in ten-year intervals, as was the age at onset of clinical signs. Additional questions addressed gender, vocational training, work experience, social life, family life, personal challenges, and ophthalmologic care.

Given the broad age range and reduced visual function of the patient cohort, the survey was conducted using a written questionnaire rather than a web-based format. Patients were contacted via postal mail using unmarked envelopes with address labels. Each mailing included:

- a cover letter explaining this study’s background, purpose, and the measures taken to ensure anonymity;
- an unmarked questionnaire;
- a prepaid, pre-addressed, unmarked return envelope.

Patients were instructed not to include any personal information, as doing so would exclude their responses from this study. Return letters were collected and opened by a designated individual (B.L.) with no access to the clinical databases. To further ensure anonymity, BL separated the questionnaires from the envelopes, removing any traceable postal documentation. A second independent evaluator (N.ZL.)—also without access to the clinical databases—analyzed the responses. To enhance anonymization, age and age at symptom onset were recorded in ten-year intervals. Patients were required to answer the first three questions regarding their age range, gender, and age at onset, while the remaining questions covering vocational training, work experience, social life, family life, personal challenges, and ophthalmologic care allowed multiple responses or the option to skip the question. All survey letters were mailed on the same day, and patients were requested to respond within a time frame of four weeks.

## 3. Results

### 3.1. Patient Characteristics

A total of 19 out of 260 survey letters were returned due to invalid addresses, resulting in a final patient cohort of 241 individuals. Within the four-week response period, 162 out of 241 patients (67.2%) submitted completed questionnaires. No responses were excluded due to the inclusion of personal information. The distributions of age groups and gender among respondents are shown in Table 1. The majority (109 out of 162; 67.3%) were under 51 years of age. In terms of gender, eighty-five respondents (52.5%) identified as female, seventy-five (46.3%) as male, and two (1.2%) as diverse. Notably, male respondents tended to be younger, with 56.0% under 41 years of age, compared to 34.1% of female respondents in the same age group.

The distribution of reported disease onset is presented in Table 2. Most patients (84 out of 162, 51.2%) experienced initial symptoms within the first two decades of life. An additional 29 patients (17.9%) reported disease onset in their third decade, while 28 patients (17.3%) noted their first symptoms after the age of 40. Gender differences were observed in the reported onset, with female patients most commonly experiencing onset in the first decade of life, whereas male patients most frequently reported onset in the second decade.

### 3.2. Vocational Training

A total of 145 out of 162 patients (89.5%) provided responses regarding their vocational training experience (Table 3). Among the 17 non-respondents, 12 were over 51 years of age. The majority of respondents (115 out of 145; 79.3%) reported no issues during their planned vocational training, with a higher percentage among females (88.0%) compared to males (70.6%). Some patients (13 out of 145; 9.0%) had not yet started vocational training, including two individuals who were unable to begin their desired program. Additionally, 14 patients (9.7%) were unable to start their intended vocational training. Six patients had to terminate their first training program (4.1%). Four patients reported a prolonged extension of more than one year for their vocational training compared to the initial plan. Two patients had to change their vocational training more than twice.

### 3.3. Professional Life

A total of 143 out of 162 patients (88.3%) provided responses regarding their professional life experiences (Table 3). Four patients who had not yet started vocational training did not respond to this question. The majority (83 out of 143; 58.0%) reported no significant difficulties in their professional careers (57.3% of females, 60.6% of males). However, thirty-eight patients (26.6%) had to retire early, including eight individuals who were unable to start their professional careers despite completing vocational training. Among patients 40 years and older, 33 out of 83 (39.8%) had to retire early. In patients 50 years and older, this proportion increased to 24 out of 48 (50.0%). Due to visual-related problems, 10 patients had to change their careers more than twice. Nineteen patients (13.3%) experienced long-term unemployment lasting more than one year.

### 3.4. Social Disadvantages

A total of 160 out of 162 patients (98.8%) provided responses regarding social disadvantages (Table 3). A minority of 31 patients (19.4%) reported experiencing no social disadvantages. However, the majority noted limitations in public life participation (106 patients; 66.3%). One-fifth complained about financial restrictions (31 patients; 19.4%). Among those reporting financial difficulties, 14 out of 38 retired early, eight out of nineteen experienced unemployment of more than a year, and four out of ten had to change careers more than twice, highlighting the impact of employment on financial independence. Additionally, 50 out of 160 patients (31.3%) received financial or other forms of support. Of these, 30 patients (60.0%) considered the support sufficient, while 20 patients (40.0%) found it insufficient, with dissatisfaction more frequently reported by female patients. Notably, 10 out of 31 patients (32.3%) who reported financial restrictions did not receive any form of financial support.

### 3.5. Family Life

A total of 131 out of 162 patients (80.9%) provided responses regarding possible disadvantages in their family life (Table 3). A vast majority, 103 out of 131 patients (78.6%), reported no negative impact of RP on their familial situation, with a slightly higher percentage among males (84.5%) compared to females (74.6%). In eight cases, RP was cited as the reason for the end of a partnership. Additionally, 24 out of 131 patients (18.3%) reported that RP influenced their decision to forgo having children. This group included five individuals who otherwise reported no disadvantages in their familial life. Patients across all age groups were affected, with 10 out of 24 being 41 years or older.

### 3.6. Personal Impairments

A total of 157 out of 162 patients (96.9%) provided responses regarding personal impairments (Table 3). Among them, only 24 patients reported feeling no personal impairments due to RP, with a significantly lower percentage among females (7.2%) compared to males (25.0%). The most frequently reported concern was anxiety about the future due to disease progression, affecting 117 out of 157 patients (74.5%). Depression was reported by 38 patients (24.2%). Additionally, 71 patients (45.2%) described experiencing other personal impairments related to RP.

### 3.7. Ophthalmic Care

A total of 154 out of 162 patients (95.0%) provided responses regarding their ophthalmological care (Table 3). A slight majority, 87 out of 154 patients (56.5%), felt that their ophthalmologic and molecular genetic diagnosis was provided within an appropriate time frame. However, even among these, 11 patients (12.6%) still perceived a delay in their RP diagnosis. A significant delay between the initial ophthalmological examination due to RP symptoms and the confirmed diagnosis was reported by 44 out of 154 patients (28.6%). Major concerns regarding the diagnostic process included a delay of more than two years in 21 patients (13.6%) and more than five years in 23 patients (14.9%). Additionally, 37 patients (24.0%) experienced one or more misdiagnoses before receiving a confirmed RP diagnosis. More than one-third of the patients, 53 out of 154 (34.4%), expressed that they lacked psychological support throughout the progression of their disease.

## 4. Discussion

To the best of our knowledge, this is the first study evaluating the patient-reported impact of RP on daily life in a cohort of genetically confirmed RP patients in Germany. The response rate of 67.2% was higher than that of a similar study in Ireland and the United Kingdom, which reported a response rate of 60% [14]. This difference may be attributed to the selection of patients receiving care in two specialized centers, the use of a short questionnaire, or the implementation of detailed anonymization measures.

Anonymization presents both advantages and disadvantages. While it may encourage participation, as evidenced by response rates ranging from 80.9 to 98.8%, it also influences response variability, with the lowest participation observed in questions related to disadvantages in family life. In a non-anonymized study, some patients might choose not to participate at all, potentially reducing the database and the diversity of responses. Whereas the present study contacted all eligible patients in a predefined consecutive series, most studies using more detailed questionnaires do not report the number of eligible patients who chose not to participate. However, a key limitation of anonymization is the inability to correlate patient responses with clinical and molecular genetic data.

A comparison with previous studies on the quality of life in RP patients must consider the significant variability in study designs. Some studies include all IRD subtypes, while others focus on RP and LCA or a specific gene without considering a specific phenotype. Additionally, there is a considerable variation in the number and detail of questions used, as well as in the correlation of findings with clinical and genetic data. Only a limited number of studies specifically include patients with molecular genetically confirmed diagnoses.

Clinical signs of RP are frequently present in the first decades of life, with most patients in this study reporting symptom onset within the first two decades. Interestingly, one-third of females reported an onset of symptoms in their first decade of life, while males most frequently reported an onset in their second decade of life. This was unexpected because x-linked RP, which predominantly affects males, is more frequent in children [21]. However, in the early stages, these impairments appear to have a limited impact on daily life, as they interfered with vocational training in only 12.9% of patients who had initiated such training. It remains unclear whether the gradual increase in visual symptoms influenced patients’ personal planning for vocational training. Research has shown that personal career aspirations do not differ significantly between individuals with and without visual impairments [22]. However, it is likely that personal experience with vision loss leads individuals to select vocational paths that are more feasible given their condition. Delayed vocational training is one of the potential factors contributing to reduced lifetime income [9].

As RP progresses, challenges in professional life become more pronounced. Some patients are unable to enter the workforce despite completing vocational training, while others are forced to leave their jobs as their condition worsens. With increasing age, the likelihood of early retirement rises, with half of the patients over 50 in this study having to retire prematurely. Given that the majority of respondents were younger than 51 years, it can be anticipated that the rate of early retirement within this group will continue to rise over time.

Extended periods of unemployment, whether due to delayed vocational training or difficulties securing employment, further contribute to reduced lifetime income [9,23]. Additionally, research has shown that visual impairment—regardless of its underlying cause—is associated with higher rates of unemployment and underemployment [24]. In recent years, rehabilitation options for visually impaired individuals have significantly improved. However, it remains to be seen whether these advancements will have a substantial impact on patients’ professional lives and long-term financial stability.

Most patients reported experiencing social disadvantages, primarily related to participation in public life (66.3%). Social isolation has been observed in 44.7–56.6% of individuals in previous studies [14,25,26]. Likely contributing factors include self-imposed limitations due to mobility difficulties, the risk of bumping into people or objects, challenges with stairs, and an increased likelihood of falls [11,26]. Participation in public life is, in addition to being impaired in syndromic RP, associated with other health problems such as Bardet–Biedl syndrome or adult Refsum disease [27,28]. Behavioral changes, such as reduced activity, have been noted even in children with visual impairment [29].

Financial restrictions were reported by 19.4% of patients, a lower percentage compared to findings from Ireland and the United Kingdom (44%) [14]. Approximately one-third of patients received financial or other forms of support, with the majority considering it adequate. However, a significant minority expressed dissatisfaction with the level of support received, and 32.3% of those reporting financial difficulties had not received any assistance. This highlights the need for a thorough evaluation and improvement of social and financial support systems to better address patients’ needs.

The extent to which the reported lack of psychological support influenced responses remains uncertain. As expected, the decline in quality of life is closely linked to the severity of visual impairment [30]. However, due to the anonymized nature of this study, no direct correlation between functional impairment and patient-reported outcomes could be established.

It is encouraging that more than 80% of patients reported no disadvantages in their family life. However, it is likely that employment and financial challenges still affected both their personal circumstances and those of their family members. Notably, one-fifth of the patients chose not to have children due to their RP. Given the age distribution of these individuals, many of these decisions were likely made before the widespread availability of detailed molecular genetic testing. This underscores the importance of timely genetic testing and appropriate counseling to support informed family planning decisions.

Anxiety and depression are frequently reported in association with IRDs [14,23,25,31,32]. In this series, nearly three-quarters of patients expressed anxiety about their future situations. Similar studies on RP and IRDs have reported anxiety rates ranging from 65.6 to 86% [11,14,33]. Detailed assessments using the verbally administered 166-item Michigan Vision-Related Anxiety Questionnaire [34] have shown that anxiety correlates with the number and severity of symptoms but not with their duration [31,35]. Consistent with these findings, the present study found that reported anxiety rates were independent of patient age or onset of symptoms. In contrast to depression, anxiety was slightly more frequent in females (78.3% vs. 69.4%). Sleeping problems associated with anxiety are prevalent in RP and appropriate psychological care might not only affect anxiety but also the quality of sleep [33].

Depression was reported by 24.2% of patients, which is lower than in a similar, non-anonymized study conducted in Ireland and the United Kingdom (65%) [14] or in patients with RP associated with adult Refsum disease (86.2%) [27]. It is important to note that these figures are based on patient-reported diagnoses without formal psychological verification. The Hospital Anxiety and Depression Scale (HADS) measures the presence of anxiety or depression within the last 7 days. In two cohorts of younger RP patients, HADS depression scores were in a similar range compared to the present study (mean age 38.2, 15.5% depression [36]; mean age 36.7, 28% depression [26]). The Beck Depression Inventory measures the severity of depression when present; moderate to severe depression was confirmed in 61% of RP patients [37]. In a nationwide study using ICD-10 coded diagnoses in newly diagnosed RP patients in Korea, the incidence of depression was 17.7% [15]. The variability of the frequency of depression is likely due to the way the diagnosis is established, as well as the criteria used to define that diagnosis. Depression is likely underdiagnosed in this population, given that many patients lack access to psychological care.

Employment and mental health appear interconnected. Patients with depression have lower employment rates [36], and unemployment is associated with higher rates of depression [32]. However, it remains challenging to determine whether RP or unemployment is the primary cause of depression or if pre-existing depression increases the risk of unemployment in RP patients. Depression itself can exacerbate financial difficulties and negatively impact patients’ social lives [38], highlighting the importance of comprehensive care that addresses both RP and mental health. Depression associated with visual impairment is common, not only in RP and IRDs but across a range of ophthalmic conditions, underscoring the need for increased attention from ophthalmologists [39,40,41]. Additionally, caregivers of IRD patients are significantly affected, with reported anxiety and depression rates of 76% and 50–78%, respectively [14].

From the patient’s perspective, ophthalmic care requires two key improvements: significantly reducing the time delay between the initial ophthalmological examination after symptom onset and clinical diagnosis and enhancing psychological support following diagnosis and throughout disease progression. In this series, 28.6% of patients reported a diagnostic delay of more than two years. Given the wide age range of participants, it is challenging to determine whether the current diagnostic process has improved compared to past experiences. Nevertheless, recent studies still highlight substantial room for improvement in diagnosing IRDs [17,42,43].

Reports of misdiagnosis should be interpreted with caution, as it can be difficult to distinguish between true diagnostic errors (e.g., mistaking RP for uveitis) and variations in terminology used by different ophthalmologists (e.g., RP, retinal dystrophy, and rod-cone dystrophy). However, the lengthy path to a definitive diagnosis suggests that many patients did, in fact, receive one or more incorrect diagnoses. To address this issue, diagnostic guidelines for IRDs were recently revised in Germany [44]. While the resources for improving patient care are in place, there is still a need to raise awareness among healthcare professionals about the importance of early and accurate IRD diagnosis. Streamlining the diagnostic process would alleviate the psychological burden associated with prolonged uncertainty.

Beyond diagnosis, continuous psychological support should be an integral part of patient care, provided by a multidisciplinary team throughout disease progression [23,45,46]. Such support could help mitigate anxiety and depression, potentially improving patients’ social interactions and work capabilities. Currently, the number of providers of psychological support experienced with IRD patients is limited, indicating the need for interdisciplinary exchange to improve patient care.

In comparison to diabetic retinopathy (DR) or age-related macular degeneration (AMD), which are more common retinal disorders potentially resulting in a marked loss of visual function, research on the quality of life has been more detailed in IRDs. In particular, data regarding the personal financial impact on patients or early retirement are limited in DR and AMD, most likely due to the older age of the patients. Anxiety (DR: 21–71%, AMD: 27–77%) and depression (DR: 14–41%, AMD 25–71%) are frequent in DR and AMD, but with a higher variability for anxiety as reported in RP [47,48,49,50,51].

The present study has several limitations. First, the inclusion of only patients with genetically confirmed RP excludes approximately 25% of RP patients for whom a genetic cause has not yet been identified. Additionally, in Germany, molecular genetic testing is more commonly initiated at specialized centers for rare retinal diseases [52]. As a result, the study population may represent individuals who either had the good fortune to access these centers or demonstrated exceptional persistence in pursuing a diagnosis, sometimes over an extended period.

Second, the anonymity of the survey introduces the possibility that some patients examined at both participating centers were contacted twice. While some of these patients may have responded only once, potentially lowering the response rate, others may have answered twice. However, given the geographical distance between the two centers, the number of such cases is likely minimal and should not significantly impact this study’s findings.

This study employed a concise questionnaire to assess the impact of RP on various aspects of patients’ lives, using six targeted questions with predefined answer choices. To preserve anonymity, the survey did not collect detailed personal comments. While this approach enabled the inclusion of a large patient cohort, it also limited the depth of individual responses.

## 5. Conclusions

The findings provide a valuable overview of the current challenges faced by patients with genetically confirmed RP in Germany. They serve as both a foundation for future research and a guide for identifying key areas requiring attention. Foremost among these is the need to enhance the diagnostic process for IRD patients. A timely and accurate diagnosis is critical, as it forms the cornerstone for initiating rehabilitation, psychological support, and financial assistance. Efforts to improve these support systems should be systematically evaluated to optimize patient care and quality of life.

Future research should focus on a more detailed analysis of the quality of life in correlation with visual function [53], the professional confirmation of anxiety and depression, and the definition of standards for psychological care in RP patients. Strategies for a multidisciplinary exchange need to be established and continuously re-evaluated to define a patient pathway beyond the initial clinical and genetic diagnosis, including a patient-focused individualized rehabilitation process and timely responses addressing social needs.

## Figures and Tables

**Table 1:** Distribution of patient age.

Age Distribution	All		Female		Male	
Years of Age	*n* = 162 *	%	*n* = 85	%	*n* = 75	%
18–20	8	4.9	2	2.4	6	8.0
21–30	32	19.8	11	12.9	21	28.0
31–40	31	19.1	16	18.8	15	20.0
41–50	38	23.5	24	28.2	13	17.3
51–60	20	12.3	15	17.7	5	6.7
61–70	28	17.3	14	16.5	13	17.3
>70	5	3.1	3	3.5	2	2.7

* Two respondents reported being divers.

**Table 2:** Distribution of patient-reported age of onset.

Onset Distribution	All		Female		Male	
Years of Age	*n* = 162 *	%	*n* = 85	%	*n* = 75	%
0–10	40	24.7	25	29.4	14	18.7
11–20	44	27.2	15	17.7	28	37.3
21–30	29	17.9	15	17.7	14	18.7
31–40	21	13.0	13	15.3	8	10.7
41–50	15	9.3	10	11.8	5	6.7
51–60	11	6.8	6	7.1	5	6.7
>61	2	1.2	1	1.2	1	1.3

* Two respondents reported being divers.

**Table 3:** Retinitis pigmentosa query results.

	All		Female		Male	
	*n* =	%	*n* =	%	*n* =	%
Contacted without return of letter	241					
Response	162	67.2	85		75	
*Vocational training response*	145	89.5	75	88.2	68	90.7
No problems	115	79.3	66	88.0	48	70.6
Not started	13	9.0	3	4.0	10	14.7
Unable to start desired training	14	9.7	4	5.3	9	13.2
Termination of desired training	6	4.1	1	1.3	4	5.9
Delay > 1 year	4	2.8	2	2.7	2	2.9
Changing more than twice	2	1.4	1	1.3	1	1.5
*Professional career responses*	143	88.3	75	88.2	66	88.0
No problems	83	58.0	43	57.3	40	60.6
Early retirement all	38	26.6	19	25.3	17	25.8
Early retirement > 40 yoa	33 (83)	39.8	18 (49)	36.7	13 (32)	40.6
Early retirement > 50 yoa	24 (48)	50.0	13 (28)	46.4	10 (19)	52.6
Changing more than twice	10	7.0	7	9.3	3	4.5
Unemployed > 1 y	19	13.3	12	16.0	7	12.1
*Social disadvantages responses*	160	98.8	85	100.0	73	97.3
None	31	19.4	11	12.9	20	27.4
Limitation in public life	106	66.3	60	70.6	44	60.3
Financial restrictions	31	19.4	19	22.4	12	16.4
Financial support acquired	50	31.3	24	28.2	26	35.6
Financial support sufficient	30 (50)	60.0	11 (24)	45.8	19 (26)	73.1
Financial support insufficient	20 (50)	40.0	13 (24)	54.2	7 (26)	26.9
*Family life disadvantages resp.*	131	80.9	71	83.5	58	77.3
None	103	78.6	53	74.6	49	84.5
IRD reason for end of partnership	8	6.1	5	7.0	2	3.4
No children due to IRD	24	18.3	14	19.7	10	17.2
*Personal impairment responses*	157	96.9	83	97.6	72	96.0
None	24	15.3	6	7,2	18	25.0
Anxiety	117	74.5	65	78.3	50	69.4
Depression	38	24.2	20	24.1	17	23.6
Other	71	45.2	41	49.4	28	38.9
*Ophthalmic care responses*	154	95.0	85	100.0	67	89.3
Appropriate time	87	56.5	44	51.8	42	62.7
Delay all	44	28.6	24	28.2	19	28.4
Delay > 2 years	21	13.6	10	11.8	10	14.9
Delay > 5 years	23	14.9	14	16.5	9	13.4
Misdiagnosis	37	24.0	21	24.7	16	23.9
Insufficient psychological support	53	34.4	28	32.9	25	37.3

## Data Availability

The data presented in this study are available on request from the corresponding author due to legal restrictions.

## Supplementary Materials

The following supporting information can be downloaded at: https://www.mdpi.com/article/10.3390/jcm14093229/s1, S1: Questionnaire: original German version, S2: Questionnaire: translated English version.

## Abbreviations

The following abbreviations are used in this manuscript:

AMD	Age-related macular degeneration
DR	Diabetic retinopathy
HADS	Hospital Anxiety and Depression Scale
IRD	Inherited retinal dystrophies
LCA	Leber congenital amaurosis
RP	Retinitis pigmentosa
